# High-performance thermochromic VO_2_-based coatings with a low transition temperature deposited on glass by a scalable technique

**DOI:** 10.1038/s41598-020-68002-5

**Published:** 2020-07-06

**Authors:** David Kolenatý, Jaroslav Vlček, Tomáš Bárta, Jiří Rezek, Jiří Houška, Stanislav Haviar

**Affiliations:** Department of Physics and NTIS – European Centre of Excellence, University of West Bohemia, Univerzitní 8, 306 14 Plzeň, Czech Republic

**Keywords:** Energy science and technology, Materials science

## Abstract

We report on high-performance thermochromic ZrO_2_/V_0.982_W_0.018_O_2_/ZrO_2_ coatings with a low transition temperature prepared on glass by a low-temperature scalable deposition technique. The V_0.982_W_0.018_O_2_ layers were deposited by a controlled high-power impulse magnetron sputtering of V target, combined with a simultaneous pulsed DC magnetron sputtering of W target to reduce the transition temperature to 20–21 °C, at a low substrate surface temperature of 330 °C in an argon–oxygen gas mixture. ZrO_2_ antireflection layers both below and above the thermochromic V_0.982_W_0.018_O_2_ layers were deposited at a low substrate temperature (< 100 °C). A coating design utilizing a second-order interference in the ZrO_2_ layers was applied to increase both the luminous transmittance (*T*_lum_) and the modulation of the solar transmittance (Δ*T*_sol_). The ZrO_2_/V_0.982_W_0.018_O_2_/ZrO_2_ coatings exhibit *T*_lum_ up to 60% at Δ*T*_sol_ close to 6% for a V_0.982_W_0.018_O_2_ thickness of 45 nm, and *T*_lum_ up to 50% at Δ*T*_sol_ above 10% for a V_0.982_W_0.018_O_2_ thickness of 69 nm.

## Introduction

Vanadium dioxide (VO_2_) undergoes a reversible phase transition from a low-temperature monoclinic VO_2_(M1) semiconductive phase to a high-temperature tetragonal VO_2_(R) metallic phase at a transition temperature (*T*_tr_) of approximately 68 °C for the bulk material^1,2^. The abrupt decrease of infrared transmittance without attenuation of luminous transmittance in the metallic state makes VO_2_-based coatings a promising candidate for thermochromic smart windows reducing the energy consumption of buildings. In spite of recent significant progress in fabrication and performance of thermochromic VO_2_-based materials (see, for example, reviews^3–8^ and the works cited therein), there are still serious drawbacks hindering their application in smart windows. These are: a high temperature needed for fabrication, a high transition temperature, a low luminous transmittance (*T*_lum_), a low modulation of the solar transmittance (Δ*T*_sol_) and low environmental stability. To meet the requirement for large-scale implementation on building glass, VO_2_-based coatings should satisfy the following criteria simultaneously: deposition temperature close to 300 °C or lower^9–13^, *T*_tr_ close to 20 °C^14^, *T*_lum_ > 60%^3,15,16^, Δ*T*_sol_ > 10%^17–19^, and long-time environmental stability^8,20–22^.

Decrease of the deposition temperature of thermochromic VO_2_-based coatings to 300 °C is of key importance: (1) to facilitate their large-scale production by reducing the energy consumption, simplifying substrate heating and cooling procedures and minimizing problems with a temperature non-uniformity over large substrate surfaces, and (2) to allow deposition of these coatings onto temperature-sensitive flexible substrates.

Magnetron sputter deposition with its versatility and the ease of scaling up to large substrate sizes is probably the most important preparation technique of thermochromic VO_2_-based coatings^8,12,13^. In our recent works^23,24^, reactive high-power impulse magnetron sputtering (HiPIMS) with an effective pulsed oxygen flow control (applicable to large-area coaters^25^) was used for low-temperature (300 °C) deposition of thermochromic VO_2_ films onto conventional soda-lime glass without any substrate bias voltage and without any interlayer. Except for the work^11^ with the same substrate surface temperature *T*_s_ = 300 °C, there is no work in the literature reporting a magnetron sputter deposition of thermochromic VO_2_ films onto unbiased amorphous substrates at *T*_s_ < 400 °C^23,24^. The possibility to prepare thermochromic VO_2_-based coatings without any substrate bias voltage is of key importance for their deposition on large area non-conductive (glass) substrates (no RF-induced bias needed). Here, it should be mentioned that HiPIMS techniques are compatible with existing magnetron sputtering systems utilized in industrial deposition devices^26,27^.

In this work, we report on high-performance three-layer thermochromic ZrO_2_/V_0.982_W_0.018_O_2_/ZrO_2_ coatings with a low transition temperature (20–21 °C) prepared on soda-lime glass using a low-temperature (330 °C) magnetron sputter deposition without any substrate bias voltage. We present basic principles of this new solution for a low-temperature scalable deposition of high-performance durable thermochromic VO_2_-based coatings for smart-window applications.

## Methods

### Coating preparation and elemental composition

**Figure 1:**
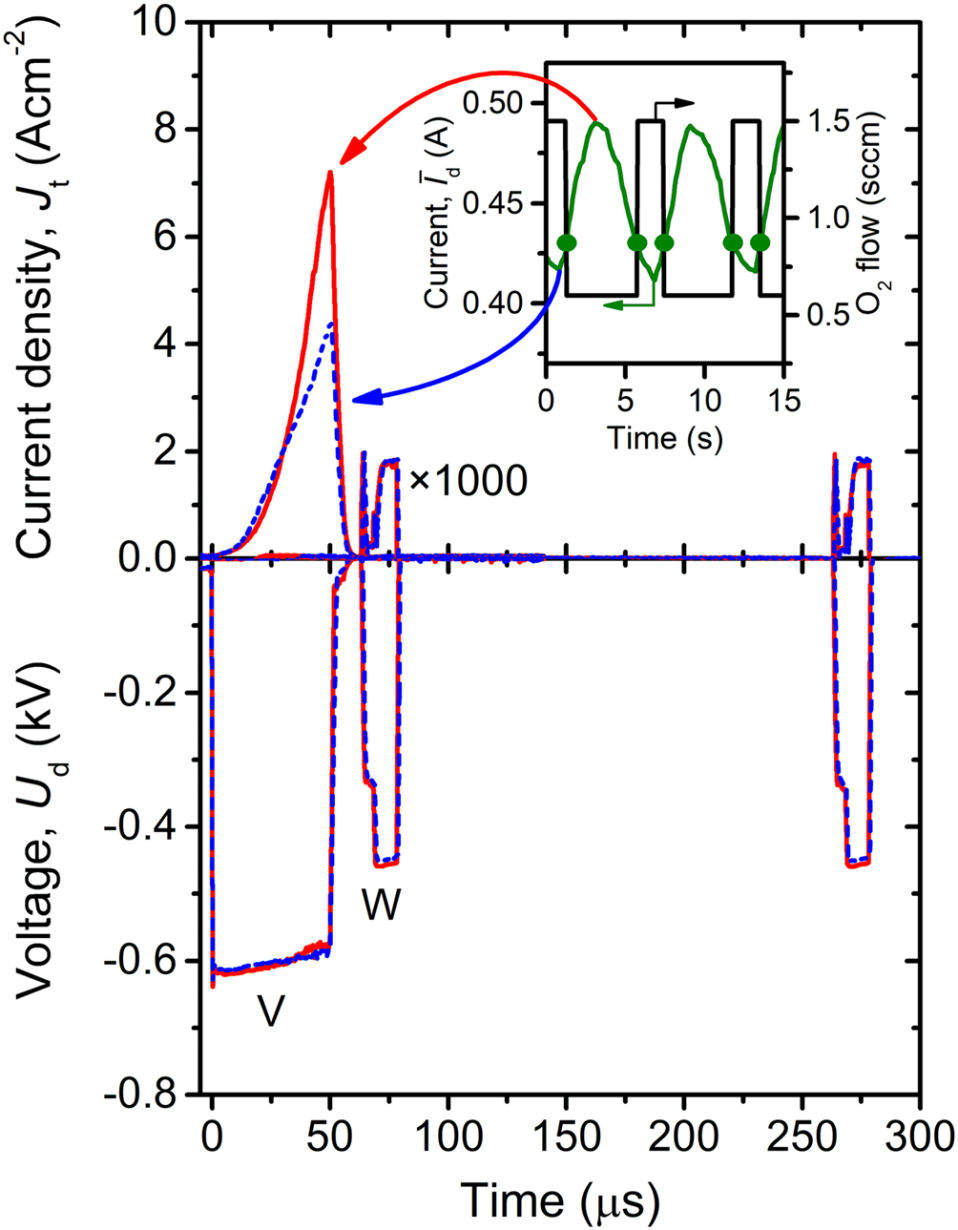
Waveforms of the magnetron voltage (*U*_d_) and the target current density (*J*_t_) for preset deposition-averaged target power densities of 12.9 W cm^−2^ and 33 mW cm^−2^ for V and W target, respectively, during a deposition of the V_0.982_W_0.018_O_2_ films (the *J*_t_ values for W target are magnified 1,000 times). Time evolution of the average discharge current on V target in a period of the power supply ($\stackrel{-}{I}$_d_) during the deposition is shown in the inset. A pre-selected critical value ($\stackrel{-}{I}$_d_)_cr_ = 0.43 A determining the switch between the oxygen flow rates *Φ*_ox_ = 0.6 sccm and *Φ*_ox_ = 1.5 sccm is marked by dots. Reprinted from the work^28^.

The V_0.982_W_0.018_O_2_ layers were deposited by controlled HiPIMS of V target, combined with a simultaneous pulsed DC magnetron sputtering of W target, at a low substrate surface temperature *T*_s_ = 330 °C and without any substrate bias voltage in an argon–oxygen gas mixture. The argon flow rate was 60 sccm corresponding to an argon partial pressure of 1 Pa, while the oxygen flow rate (*Φ*_ox_) was not fixed but pulsing between 0.6 and 1.5 sccm (see Fig. 1), and the duration of the *Φ*_ox_ pulses (injecting oxygen in front of the sputtered V target toward the substrate^24^) was determined during the deposition by a programmable logic controller^29^ using a pre-selected critical value of the average discharge current on V target in a period of the power supply ($\stackrel{-}{I}$_d_)_cr_ = 0.43 A. The basic principle of the pulsed oxygen flow control is illustrated in Fig. 1, which shows the time evolution of the magnetron voltage (*U*_d_(t)) and the target current density (*J*_t_(t)), averaged over the total target area, for both targets at the minimum and maximum value of the oxygen partial pressure in the vacuum chamber corresponding to the minimum and maximum $\stackrel{-}{I}$_d_, respectively, during the deposition. Here, it should be mentioned that the effective pulsed oxygen flow control makes it possible to utilize two benefits of the reactive HiPIMS deposition^23,24^. The first benefit is highly ionized fluxes of particles with many V^+^ and V^2+^ ions onto the substrate and enhanced energies (up to 50 eV relative to ground potential) of the ions bombarding the growing films, allowing us to achieve the VO_2_ crystallinity at a low *T*_s_ and without any substrate bias voltage. The second benefit is a very high degree of dissociation of the O_2_ molecules injected into the high-density plasma in front of the V target, allowing us to achieve the required VO_2_ stoichiometry at a low compound fraction in the target surface layer. This is of key importance for reduced arcing, increased sputtering of V atoms, and low production of O^−^ ions at the target^29^.

The depositions were performed in an ultra-high vacuum multi-magnetron sputter device (ATC 2200-V AJA International Inc.) using two unbalanced magnetrons with planar V and W targets (99.9% purity, diameter of 50 mm and thickness of 6 mm in both cases). The magnetron with a V target was driven by a high-power pulsed DC power supply (TruPlasma Highpulse 4002 TRUMPF Huettinger)^24^. The voltage pulse duration was 50 µs at a repetition frequency of 200 Hz (duty cycle of 1%) and the deposition-averaged target power density was 12.9 W cm^−2^. The magnetron with a W target was driven by a pulsed DC power supply (IAP-1010 EN Technologies Inc.). The voltage pulse duration was 16 µs at a repetition frequency of 5 kHz (duty cycle of 8%) and the deposition-averaged target power density was 33 mW cm^−2^. Under these conditions, the W content in the metal sublattice of V_1−*x*_W_*x*_O_2_, as measured on a dedicated 285 nm thick film in a scanning electron microscope (SU-70, Hitachi) using wave-dispersive spectroscopy (Magnaray, Thermo Scientific), was 1.8 ± 0.6 at.% (i.e., *x* = 0.018).

Both bottom and top ZrO_2_ antireflection layers were deposited by reactive mid-frequency AC magnetron sputtering without ohmic heating (*T*_s_ < 100 °C) and without any substrate bias voltage in an argon–oxygen gas mixture. The argon partial pressure was 1 Pa and the oxygen partial pressure was 0.35 Pa (oxide mode). The depositions were performed using two strongly unbalanced magnetrons with planar Zr targets (99.9% purity, diameter of 100 mm and thickness of 6 mm) driven by a mid-frequency AC power supply (TruPlasma MF 3010, TRUMPF Huettinger)^30^. The oscillation frequency was close to 85 kHz and the deposition-averaged target power density was 15.5 W cm^−2^.

The thickness of individual layers was measured by spectroscopic ellipsometry using the J. A. Woollam Co. Inc. VASE instrument^31^.

The presented deposition technique for preparation of the thermochromic ZrO_2_/V_0.982_W_0.018_O_2_/ZrO_2_ coatings is, just like the deposition of low-emissivity coatings, compatible with the existing magnetron sputtering systems in glass production lines.

### Coating structure and properties

For structural investigation of the films, X-ray diffraction (XRD) measurements were carried out using a PANalytical X´Pert PRO diffractometer working with a CuKα (40 kV, 40 mA) radiation at a glancing incidence of 1°. The average size of coherently diffracting regions of the VO_2_(R)/VO_2_(M1) phase was estimated from the full width at half maximum of the main VO_2_(R)/VO_2_(M1) diffraction peak, corrected for instrumental broadening, using the Scherrer’s equation.

The surface morphology of the films was determined by atomic force microscopy (AFM) using a SmartSPM Microscope (AIST-NT) with a diamond tip (nominal radius below 10 nm) in a semicontact mode. The root-mean-square roughness of the surface, *R*_rms_, was computed from a randomly selected square area of 1 × 1 μm^2^. The AFM images were processed by Gwyddion 2.41 software^32^, and an implemented “watershed” method was used for grain analysis. The grains identified were approximated by an equivalent disc diameter with the same projected area as the grain.

The hardness of VO_2_ (without the ZrO_2_ overlayer) was measured using a Hysitron TI 950 triboindenter with a cube corner tip at a maximum load of 100 μN.

The normal-incidence coating transmittance was measured by spectrophotometry using the Agilent CARY 7000 instrument equipped with an in-house made heat/cool cell. Spectroscopic measurements were performed in the wavelength range *λ* = 300 to 2,500 nm at the temperatures *T*_ms_ = − 5 °C (semiconducting state below *T*_tr_) and *T*_mm_ = 60 °C (metallic state above *T*_tr_). Hysteresis curves were measured at *λ* = 2,500 nm in the temperature range *T*_m_ = − 10 to 60 °C. The luminous transmittance (*T*_lum_) and the solar transmittance (*T*_sol_) are defined as follows

1 $${T}_{\mathrm{l}\mathrm{u}\mathrm{m}}\left({T}_{\mathrm{m}}\right)=\frac{\underset{380}{\overset{780}{\int }}{\varphi }_{\mathrm{l}\mathrm{u}\mathrm{m}}{\left(\lambda \right)\varphi }_{\mathrm{s}\mathrm{o}\mathrm{l}}\left(\lambda \right)T\left(\lambda , {T}_{\mathrm{m}}\right)d\lambda }{\underset{380}{\overset{780}{\int }}{\varphi }_{\mathrm{l}\mathrm{u}\mathrm{m}}{\left(\lambda \right)\varphi }_{\mathrm{s}\mathrm{o}\mathrm{l}}\left(\lambda \right)d\lambda },$$

2 $${T}_{\mathrm{s}\mathrm{o}\mathrm{l}}\left({T}_{\mathrm{m}}\right)=\frac{\underset{300}{\overset{2500}{\int }}{\varphi }_{\mathrm{s}\mathrm{o}\mathrm{l}}\left(\lambda \right)T\left(\lambda , {T}_{\mathrm{m}}\right)d\lambda }{\underset{300}{\overset{2500}{\int }}{\varphi }_{\mathrm{s}\mathrm{o}\mathrm{l}}\left(\lambda \right)d\lambda },$$

where *φ*_lum_ is the luminous sensitivity of the human eye and *φ*_sol_ is the sea-level solar irradiance spectrum^33^ at an air mass of 1.5. The modulation of the luminous transmittance (Δ*T*_lum_) and of the solar transmittance (Δ*T*_sol_) are defined as

3 $$\Delta {T}_{\mathrm{l}\mathrm{u}\mathrm{m}}= {T}_{\mathrm{l}\mathrm{u}\mathrm{m}}\left({T}_{\mathrm{m}\mathrm{s}}\right)- {T}_{\mathrm{l}\mathrm{u}\mathrm{m}}\left({T}_{\mathrm{m}\mathrm{m}}\right),$$

4 $$\Delta {T}_{\mathrm{s}\mathrm{o}\mathrm{l}}= {T}_{\mathrm{s}\mathrm{o}\mathrm{l}}\left({T}_{\mathrm{m}\mathrm{s}}\right)- {T}_{\mathrm{s}\mathrm{o}\mathrm{l}}\left({T}_{\mathrm{m}\mathrm{m}}\right).$$

Using relation (2) it can be written

5 $${\Delta T}_{\mathrm{s}\mathrm{o}\mathrm{l}}=\frac{\underset{300}{\overset{2500}{\int }}{\varphi }_{\mathrm{s}\mathrm{o}\mathrm{l}}\left(\lambda \right)\Delta T\left(\lambda \right)d\lambda }{\underset{300}{\overset{2500}{\int }}{\varphi }_{\mathrm{s}\mathrm{o}\mathrm{l}}\left(\lambda \right)d\lambda },$$

where Δ*T*(*λ*) = *T*(*λ*, *T*_ms_) − *T*(*λ*, *T*_mm_) is the modulation of the transmittance at the wavelength *λ*. The average luminous transmittance (*T*_lum_) is defined as *T*_lum_ = [*T*_lum_(*T*_ms_) + *T*_lum_(*T*_mm_)]/2.

## Results and discussion

### Design and transition temperature of ZrO_2_/V_0.982_W_0.018_O_2_/ZrO_2_ coatings

**Figure 2:**
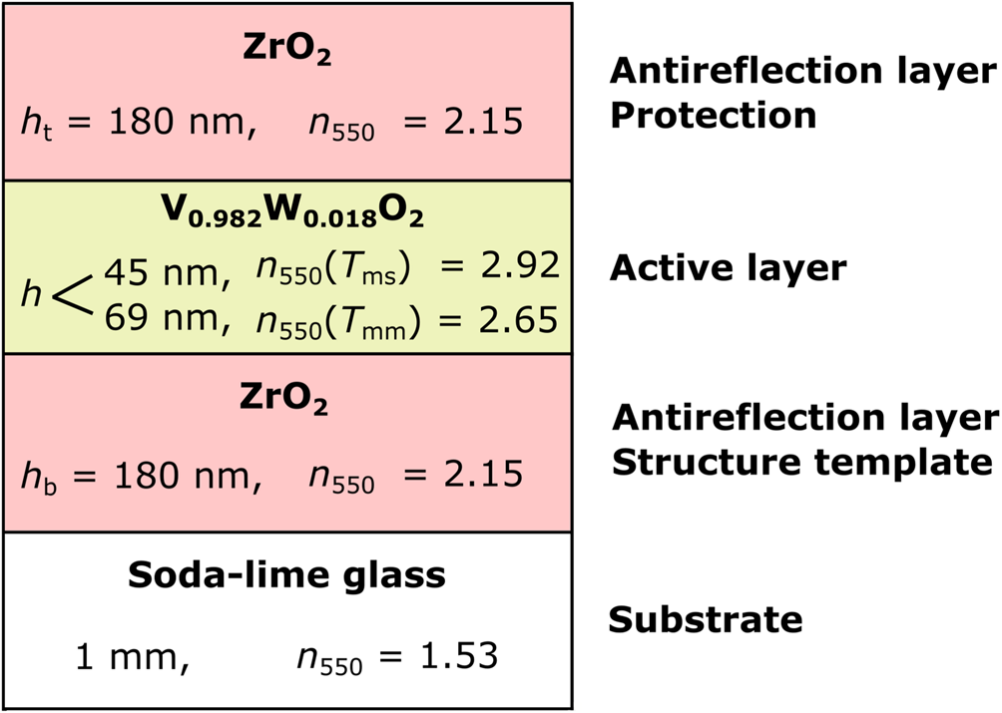
The three-layer thermochromic VO_2_-based coating on a soda-lime glass substrate investigated in this paper. Here, *h*_b_, *h*, and *h*_t_ represent the thickness of the bottom ZrO_2_ layer, the thickness of the active V_0.982_W_0.018_O_2_ layer, and the thickness of the top ZrO_2_ layer, respectively. Below, individual coatings are referred to as (*h*_b_, *h*, *h*_t_). The refractive index (*n*_550_) at the wavelength of 550 nm of all layers is also given. *T*_ms_ and *T*_mm_ denote the temperatures when the V_0.982_W_0.018_O_2_ layer is in the semiconductive (below *T*_tr_) and metallic (above *T*_tr_) state, respectively. Reprinted from the work^28^.

The three-layer structure of ZrO_2_/V_0.982_W_0.018_O_2_/ZrO_2_ coatings, formed by an active layer in the middle and two antireflection (AR) layers, is shown in Fig. 2. Let us emphasize the combination of properties which makes ZrO_2_ a proper candidate for the AR-layers. First, ZrO_2_ has a refractive index (*n*) close to the required geometric mean of refractive indices of V_0.982_W_0.018_O_2_ and glass (bottom AR-layer) or V_0.982_W_0.018_O_2_ and air (top AR-layer). Second, ZrO_2_ has almost zero extinction coefficient (*k*) for visible and infrared wavelengths (*λ*), allowing one to utilize higher-order AR-layers without concessions in terms of absorption. Third, crystalline structure of the bottom ZrO_2_ layer can be achieved even at a low deposition temperature, which in turn improves the V_0.982_W_0.018_O_2_ crystallinity and the process reproducibility. Fourth, ZrO_2_ is a hard (for an oxide) and stable material, which allows the top AR-layer to provide a mechanical protection and environmental stability for the active V_0.982_W_0.018_O_2_ layer. The hardness of ZrO_2_ prepared by the present technique is 15–17 GPa^30^, compared to the hardness of VO_2_ of only 12 GPa. Note that ZrO_2_ layers are being increasingly applied in architectural glass as a protective overcoat for advanced low-emissivity stacks^34^. These properties cannot be matched by many other potential or occasionally used AR-layer materials due to their, e.g., non-zero *k* (Cr_2_O_3_), lower hardness (SiO_2_, Ta_2_O_5_), high deposition temperature of the hardest phase (α-Al_2_O_3_), too low *n* for the bottom AR-layer (SiO_2_) or usable but too high *n* (rutile TiO_2_).

We examined the effect of smoothly varied *h*_b_ and *h*_t_ in our recent work^31^ and thereby identified the optimum value *h*_b_ = *h*_t_ = 180 nm leading to a second-order interference maximum of *T*_lum_ (consistently with the optimization of *h*_t_ in the work^35^). This choice constitutes a crucial part of the efforts to maximize *T*_lum_ and Δ*T*_sol_ (at a given *h*) in parallel: while the frequently used first-order AR-layers (*λ*/4-layers; see e.g. the work^36^ for a first-order ZrO_2_ AR-layer) lead to a high transmittance modulation only in the far infrared (where it is weighted by weak solar irradiance when calculating Δ*T*_sol_), second-order AR-layers (3*λ*/4-layers) lead to a high transmittance modulation mainly in the near infrared (where it is weighted by much higher solar irradiance; see below for a graphical example). Furthermore, we use two different *h* values of 45 nm or 48 nm (leading to higher *T*_lum_) and 69 nm (leading to higher Δ*T*_sol_) in order to demonstrate the corresponding tradeoff. Here, it should be mentioned that the thickness of the V_0.982_W_0.018_O_2_ layer deposited onto amorphous soda-lime glass was 48 nm while it was 45 nm for the same layer deposited using the same discharge conditions (Fig. 1) and deposition time onto the crystalline ZrO_2_ layer. To avoid any changes in the composition of the V_0.982_W_0.018_O_2_ layer, which would be caused mainly by a larger erosion of the V target (see the much higher power compared with the W target) in additional (much later) depositions, we used the original configurations of the thermochromic VO_2_-based coatings with the slightly different *h* = 45 nm and 48 nm in this work. The aforementioned effect of the thicker V_0.982_W_0.018_O_2_ layer with *h* = 69 nm is presented for two important configurations, denoted as (180, 69, 0) and (180, 69, 180).

**Figure 3:**
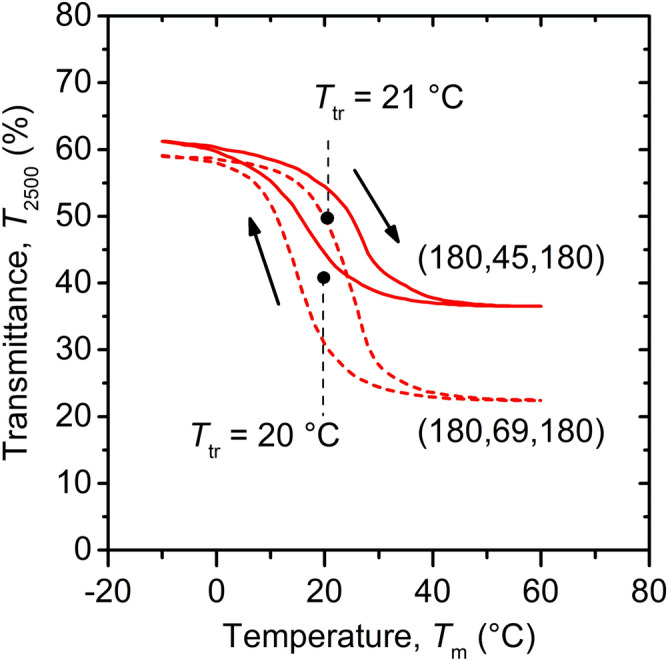
Temperature (*T*_m_) dependence of the transmittance (*T*_2500_) at 2,500 nm for the ZrO_2_/V_0.982_W_0.018_O_2_/ZrO_2_ coatings with *h*_b_ = 180 nm, *h* = 45 nm or 69 nm, and *h*_t_ = 180 nm deposited onto 1 mm thick glass substrates (Fig. 2). The transition temperatures (*T*_tr_) are also given. Adapted from the work.^28^

Figure 3 shows the dependence of the transmittance at *λ* = 2,500 nm (*T*_2500_) on the measurement temperature (*T*_m_) for two of the coatings prepared. The evaluated transition temperature (center of the hysteresis curves) was reduced by the aforementioned W doping to *T*_tr_ = 20–21 °C (ZrO_2_/V_0.982_W_0.018_O_2_/ZrO_2_ coatings in Fig. 2) or 23 °C (V_0.982_W_0.018_O_2_ without AR-layers in Table 1). The *T*_tr_ value is reproducible (almost the same for two different *h* values) and in agreement with the requirement for smart-window applications^14^. It is very important that using the present deposition technique, we did not experience any tradeoff (indicated in the literature^19,37,38^) between lowering *T*_tr_ by W doping and optimizing the other optical properties: the differences in the V(W)O_2_ optical constants at *λ* = 550 nm were within the measurement error and reproducibility noise and did not exhibit any systematic dependence on the W content. The present W content of 1.8 ± 0.6 at.% and the transition temperature *T*_tr_ = 57 °C achieved for undoped VO_2_ prepared by the same technique^23^ collectively lead to a gradient of approximately (23–57)/1.8 = − 19 K/at.% of W in the metal sublattice, consistent with those (− 13 to − 22 K/at.%) reported in the literature^18,31,39^.

**Table 1:** Thermochromic properties of different configurations of the VO_2_-based coatings on 1 mm thick glass substrates.

*h* (nm)	*h*_b_ (nm)	*h*_t_ (nm)	*T*_tr_ (°C)	*T*_lum_ (*T*_ms_) (%)	*T*_lum_ (*T*_mm_) (%)	Δ*T*_lum_ (%)	*T*_sol_ (*T*_ms_) (%)	*T*_sol_ (*T*_mm_) (%)	Δ*T*_sol_ (%)
48	0	0	23	33.5	35.5	− 2.0	34.6	31.7	2.9
45	180	0	20	41.8	43.1	− 1.3	40.4	36.1	4.3
69	180	0	19	38.6	36.6	2.1	34.4	26.3	8.1
48	0	180	25	54.3	53.5	0.8	46.3	41.8	4.5
45	180	180	21	59.7	59.1	0.6	49.9	44.4	5.5
69	180	180	20	49.9	46.0	3.9	42.4	32.0	10.4

Here, *h* is the thickness of the V_0.982_W_0.018_O_2_ layer, and *h*_b_ and *h*_t_ are thicknesses of the bottom and top ZrO_2_ layers, respectively. Adapted from the work^28^.

### Structure and morphology of V_0.982_W_0.018_O_2_ layers

**Figure 4:**
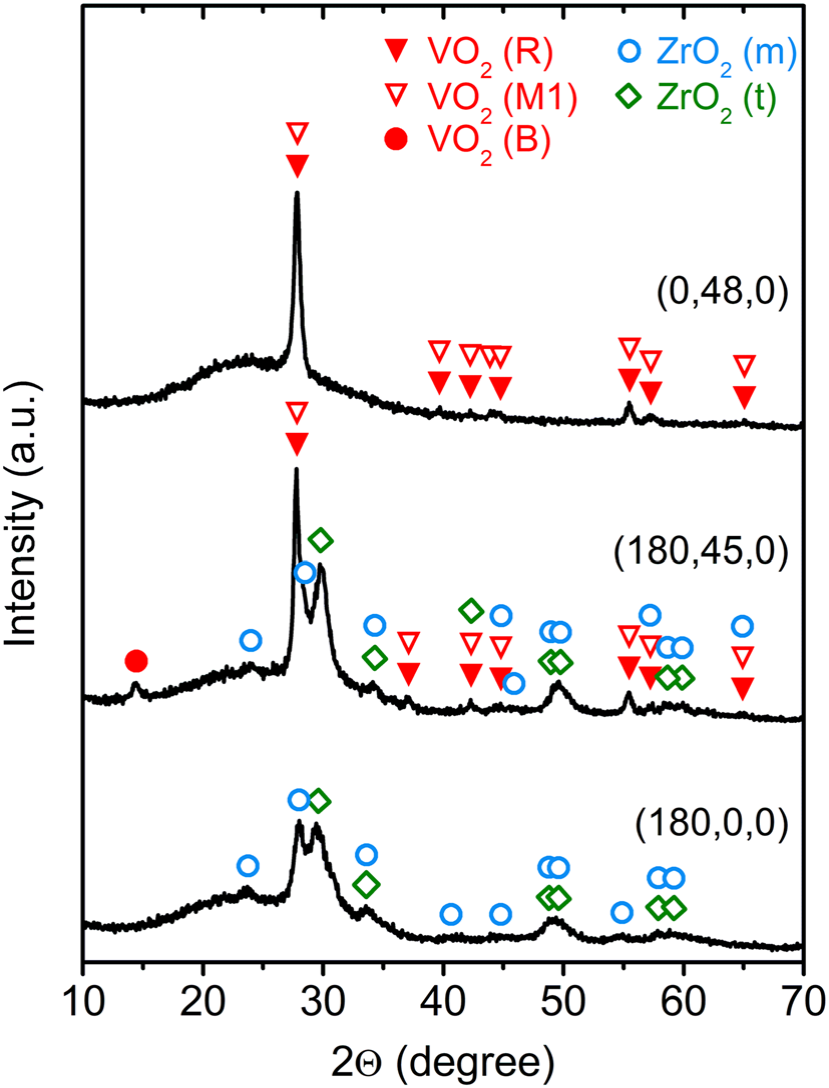
X-ray diffraction patterns taken at *T*_m_ = 25 °C from the V_0.982_W_0.018_O_2_ layers with the thickness *h* = 48 nm deposited onto glass [denoted as (0,48,0)] and with the thickness *h* = 45 nm deposited under the same discharge conditions (Fig. 1) onto the ZrO_2_ layer with the thickness *h*_b_ = 180 nm on glass [denoted as (180,45,0)]. For comparison, the X-ray diffraction pattern from the ZrO_2_ layer with the thickness *h*_b_ = 180 nm on glass [denoted as (180,0,0)] is given. The main diffraction peaks of VO_2_(R), VO_2_(M1), VO_2_(B), ZrO_2_(m) and ZrO_2_(t) are marked. Reprinted from the work^28^.

Figure 4 shows the coating structure at the temperature *T*_m_ = 25 °C, i.e., essentially during the thermochromic transition. The bottom ZrO_2_ AR-layer [denoted as (180,0,0)] consists of a mixture of m-ZrO_2_ (PDF#04-013-6875^40^) and t-ZrO_2_ (PDF#01-081-1544 valid for ZrO_1.95_). The strongest peaks around 2Θ = 28.0° and 29.5° correspond well to the peaks of m-ZrO_2_ [(− 111) planes diffracting at 2Θ = 27.95°] and t-ZrO_2_ [(101) planes diffracting at 2Θ = 29.81°]. The V_0.982_W_0.018_O_2_ layer with *T*_tr_ = 23 °C (Table 1) deposited onto glass [denoted as (0,48,0)] and the V_0.982_W_0.018_O_2_ layer with *T*_tr_ = 20 °C deposited onto the crystalline ZrO_2_ layer [denoted as (180,45,0)] consist of a mixture of the high- and low-temperature thermochromic phase, VO_2_(R) (PDF#01-073-2362) and VO_2_(M1) (PDF#04-003-2035), respectively, which are hard to distinguish. The strongest and sharp peak around 2Θ = 27.8° corresponds well to the peaks of the VO_2_(R), (110) planes diffracting at 2Θ = 27.91°, and the VO_2_(M1), (011) planes diffracting at 2Θ = 27.80°. In spite of a small content of the non-thermochromic VO_2_(B) phase (PDF#01-084-3056), the V_0.982_W_0.018_O_2_ layer on the crystalline ZrO_2_ layer exhibits a better crystallinity of the thermochromic VO_2_(R)/VO_2_(M1) phase. This is quantified in terms of larger size of the coherently diffracting regions, obtained using the peak around 27.8°. The size is 47 nm along the scattering vector (that is, for the 1° glancing incidence used, about 47·cos[1° + 27.8°/2] = 45 nm vertically, which is equal to the layer thickness) for the V_0.982_W_0.018_O_2_ layer on ZrO_2_, compared to 23 nm for the V_0.982_W_0.018_O_2_ layer on glass.

**Figure 5:**
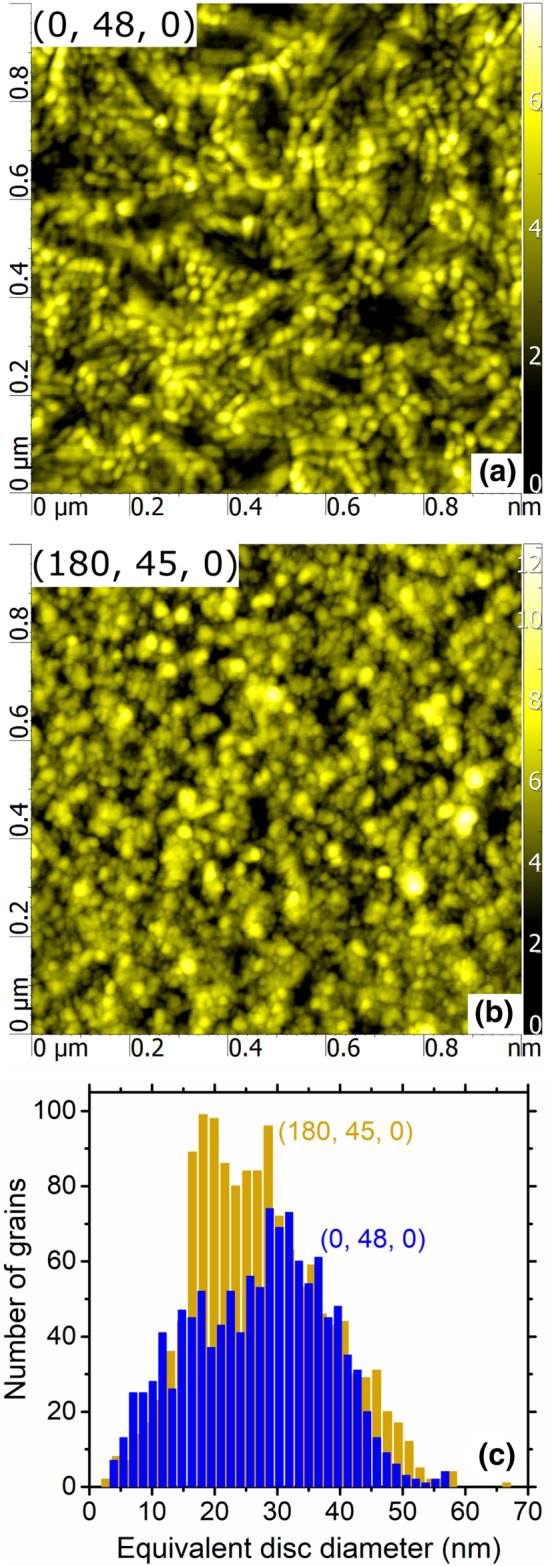
Surface morphology of the V_0.982_W_0.018_O_2_ layers with the thickness *h* = 48 nm deposited onto glass [denoted as (0,48,0); panel **a**] and with the thickness *h* = 45 nm deposited onto the ZrO_2_ layer with the thickness *h*_b_ = 180 nm on glass [denoted as (180,45,0); panel **b**], together with the corresponding grain-size (approximated by an equivalent disc diameter) distributions on the area of 1 × 1 μm^2^; panel **c**. Reprinted from the work^28^.

The surface morphology of V_0.982_W_0.018_O_2_ layers (without the top AR-layer) is shown in Fig. 5. The figure constitutes an independent confirmation of the fact that while the present deposition technique allows a low-temperature crystallization of VO_2_-based layers on amorphous glass, their crystallinity on crystalline ZrO_2_ is even better. Most importantly, the grains identified by the “watershed” method make up 80% of the projected surface area for the V_0.982_W_0.018_O_2_ layer (*R*_rms_ = 1.1 nm) deposited onto the bare soda-lime glass (Fig. 5a), while they make up 94% of the projected surface area for the V_0.982_W_0.018_O_2_ layer (*R*_rms_ = 1.2 nm) deposited onto the crystalline ZrO_2_ AR-layer (Fig. 5b). Furthermore, the latter V_0.982_W_0.018_O_2_ layer exhibits also a narrower distribution of the horizontal grain sizes (Fig. 5c).

### Thermochromic properties of ZrO_2_/V_0.982_W_0.018_O_2_/ZrO_2_ coatings

**Figure 6:**
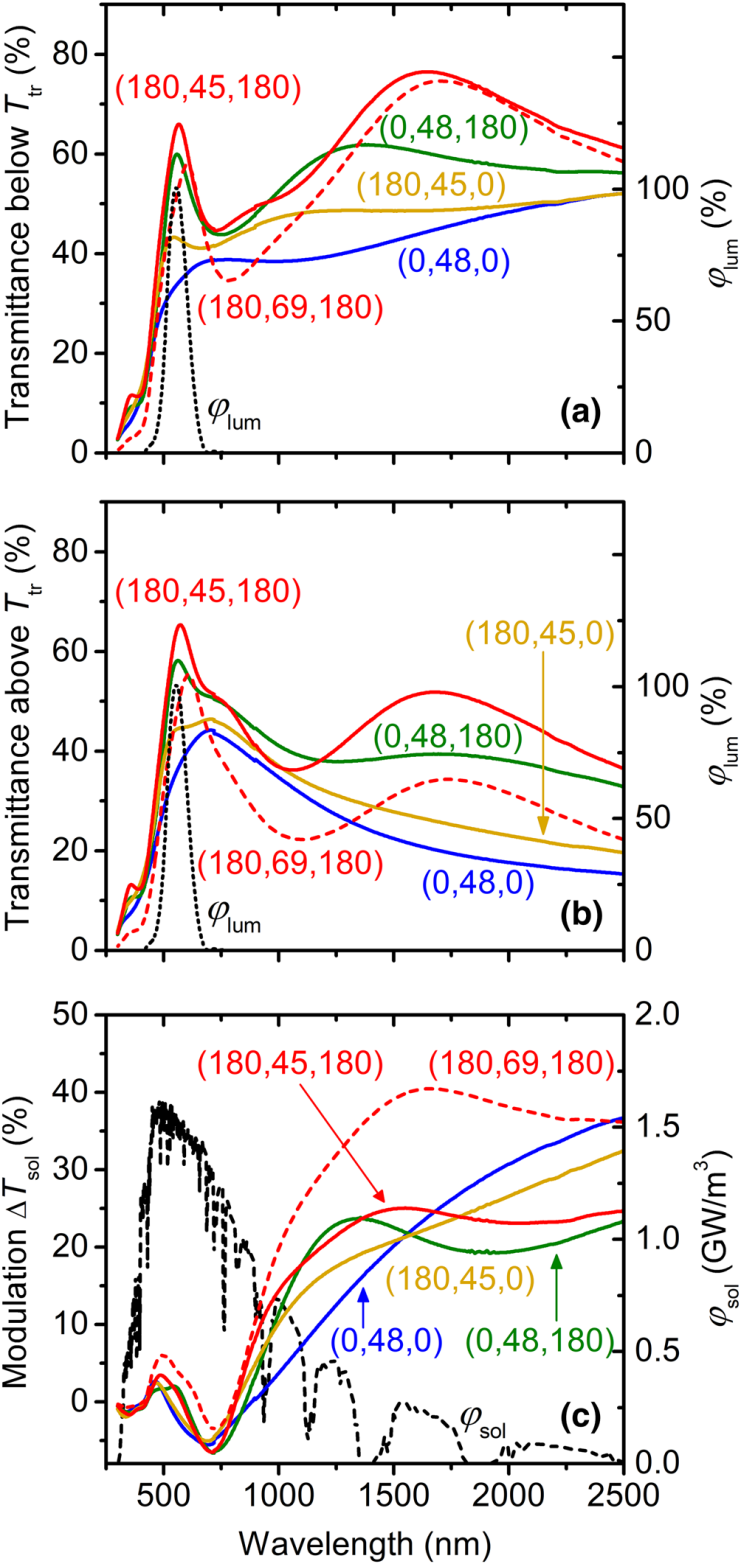
Spectral transmittance *T*(*λ, T*_m_) of different configurations (Table 1) of the thermochromic VO_2_-based coatings on 1 mm thick glass substrates measured at *T*_ms_ = − 5 °C (panel **a**) and *T*_mm_ = 60 °C (panel **b**), together with the corresponding modulation of the transmittance Δ*T*(*λ*); panel **c**. The coatings are denoted as (*h*_b_, *h*, *h*_t_), where *h*_b_ and *h*_t_ are the thicknesses of the bottom and top ZrO_2_ layers, respectively. The thickness of the V_0.982_W_0.018_O_2_ layer is *h* = 45 nm or 48 nm (solid lines) and *h* = 69 nm (dashed lines). The luminous sensitivity of the human eye (*φ*_lum_) normalized to a maximum of 100%, and the sea-level solar irradiance spectrum (*φ*_sol_) at an air mass of 1.5 are also given. Adapted from the work.^28^

Figure 6 shows in detail the aforementioned role of second-order AR-layers in optimizing *T*_lum_ and Δ*T*_sol_, given by Eqs. (1) and (5), respectively, in parallel. On the one hand, Fig. 6a,b show that *T*_lum_ depends only on a narrow range of wavelengths: the transmittance *T*(*λ, T*_m_) is weighted by a narrow function *φ*_lum_(*λ*). There is an easily explainable increase of *T*(*λ, T*_m_) in the corresponding narrow visible *λ* range, resulting from using only the bottom AR-layer, only the top AR-layer (stronger increase than the previous one) and both AR-layers (the strongest increase). This phenomenon is almost independent of *T*_m_, which means that the low Δ*T*_lum_ (Table 1) is almost independent of the coating design. Furthermore, Fig. 6a,b confirm that owing to the absorption in V_0.982_W_0.018_O_2_, *T*(*λ, T*_m_) is generally higher at *h* = 45 nm and 48 nm than at *h* = 69 nm.

On the other hand, Fig. 6c shows that the dependence of Δ*T*_sol_ on the coating configuration is much more difficult to explain, because the transmittance modulation Δ*T*(*λ*) is weighted by a wide and complicated function *φ*_sol_(*λ*) and there is no coating configuration leading to the highest Δ*T*(*λ*) in the whole *λ* range shown. Indeed, while Δ*T*(*λ*) in the far infrared above ≈1,600 nm (weighted by relatively low *φ*_sol_) is actually the highest without any AR-layer, Δ*T*(*λ*) in the near infrared below ≈1,600 nm (weighted by relatively high *φ*_sol_) is the highest when using both second-order AR-layers or at least the top one. The reason is that the second-order interference maxima on both AR-layers in the visible are followed by lower-order interference minima and maxima in the infrared, and that the overall improvement of the near infrared transmittance by this interference is more significant below than above *T*_tr_. The fact that this kind of effect cannot be achieved by thinner first-order AR-layers is discussed in more detail in our recent work^31^. Furthermore, Fig. 6c confirms that Δ*T*(*λ*) is generally higher at *h* = 69 nm than at *h* = 45 nm or 48 nm.

**Figure 7:**
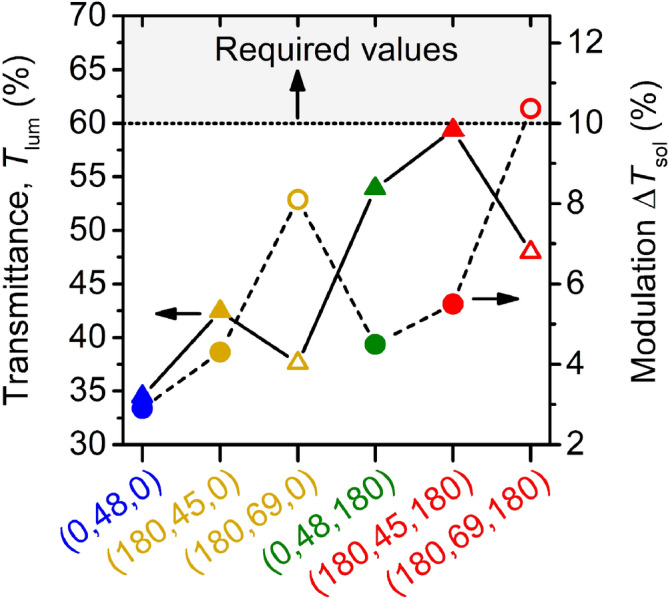
The average luminous transmittance (*T*_lum_) and the modulation of the solar transmittance (Δ*T*_sol_) for different configurations (Table 1) of the thermochromic VO_2_-based coatings on 1 mm thick glass substrates. The coatings are denoted as (*h*_b_, *h*, *h*_t_), where *h*_b_ and *h*_t_ are thicknesses of the bottom and top ZrO_2_ layers, respectively. The thickness of the V_0.982_W_0.018_O_2_ layer is *h* = 45 nm or 48 nm (full symbols) and *h* = 69 nm (empty symbols). The gray region represents the required values for smart-window applications. Adapted from the work.^28^

The transmittance-based integral quantities, given by Eqs. (1)–(5), are summarized in Fig. 7 and Table 1. In agreement with the discussion of the transmittance in itself (Fig. 6), it can be seen that the transition from (1) bare V_0.982_W_0.018_O_2_ through coatings with (2) only the bottom AR-layer and (3) only the top AR-layer to the coating with (4) both AR-layers, at almost the same thickness (45 nm and 48 nm) of the V_0.982_W_0.018_O_2_ layer, leads to a gradual improvement of the optical performance (average *T*_lum_ and Δ*T*_sol_). The performance of the best coating configuration (*h*_b_ = *h*_t_ = 180 nm) is characterized by *T*_lum_ = 59.4% and Δ*T*_sol_ = 5.5% (at *h* = 45 nm) and by *T*_lum_ = 48.0% and Δ*T*_sol_ = 10.4% (at *h* = 69 nm), in both cases accompanied by low Δ*T*_lum_ and the aforementioned *T*_tr_ = 20–21 °C. It is possible to state that our results are close to the requirements (see the introductory part and the gray area in Fig. 7) for smart-window applications.

In addition to comparing the thermochromic properties of the present coatings with the industrial requirements, it is worth comparing them with the properties of coatings reported in the literature (Table 2). We focus on coatings on glass substrates and on plastic foils^42,43^, which can be pasted on the glass, with an at least somewhat lowered *T*_tr_ ≤ 40 °C.

**Table 2:** Comparison between this work and previously reported studies on *T*_lum_ and Δ*T*_sol_ of VO_2_-based coatings with a transition temperature *T*_tr_ ≤ 40 °C prepared on glass substrates or polyethylene terephthalate (PET) foils.

(*T*_s_)_max_ (°C)	*T*_tr_ (°C)	*T*_lum_ (*T*_ms_) (%)	*T*_lum_ (*T*_mm_) (%)	Δ*T*_sol_ (%)	*h* (nm)	Preparation method	Refs.
Substrates: glass, silica* and fused quartz^**+**^							
330	20	49.9	46.0	10.4	69	HiPIMS + ACMS	This work
330	21	59.7	59.1	5.5	45	HiPIMS + ACMS	This work
330	40	43.9	40.0	11.6	76	HiPIMS + ACMS	^31^
400	32	60.9	65.0	3.6	Nanop.	Hydroth. + Anneal.	^17^
450	29	61.8	61.0	5.2	100	RFMS	^18^
450	34	36.2	32.3	14.6	145	DCMS + Anneal.	^19^
450	38	45.2	39.0	12.8	150	DCMS + Anneal.	^37^
500*	28	44.6	45.7	6.9	100	Sol–Gel + Anneal.	^15^
600^+^	35	71.6	70.1	8.6	392	Sol–Gel + Anneal.	^41^
**Substrate: PET foils**							
50	35	48.7	45.9	10.7	Nanop.	Hydroth. + Disper.	^42^
100	36	56.0	–	12.7	Nanop.	Hydroth. + Disper.	^43^

(*T*_s_)_max_ is the maximum substrate temperature during the preparation of the coatings and *h* is the thickness of the active VO_2_-based layer. Here, ACMS, RFMS and DCMS denote the AC, RF and DC magnetron sputtering, respectively.

The ZrO_2_/V_0.988_W_0.012_O_2_/ZrO_2_ coating^31^ was deposited using the same method as in the present work. The V_0.958_Tb_0.031_W_0.011_O_2_ coating^17^ was fabricated on a glass substrate from Tb- and W-codoped VO_2_ nanopowders prepared using hydrothermal synthesis. An additional annealing at 400 °C for 60 min in argon atmosphere was ultimately applied to increase the adhesion and the coating crystallinity. The V_0.872_Sr_0.119_W_0.009_O_2_ layer, forming a basis for the V_0.872_Sr_0.119_W_0.009_O_2_/SnO_2_ coating^18^, was deposited by RF magnetron co-sputtering of V, Sr and W from a single composed V-Sr-W target in an argon–oxygen gas mixture at a substrate temperature of 450 °C. The V_0.931_Fe_0.069_O_2_ coating^19^ and V_0.878_Fe_0.092_Mg_0.030_O_2_ coating^37^ were prepared by DC magnetron co-sputtering of V and Fe, and V, Fe and Mg, respectively, from a single composed V–Fe, and V–Fe–Mg target in an argon–oxygen gas mixture at a substrate temperature of 60 °C, with an additional in-situ annealing at 450 °C for 30 min in oxygen. The V_0.98_W_0.02_O_2_ coating^15^ was prepared on silica substrate by spin coating via a sol–gel process and annealing at 500 °C for 30 min in ammonia atmosphere. The V_0.99_W_0.01_O_2_ coating^41^ was prepared on fused quartz using a sol–gel method and annealing at 600 °C for 30 min in argon gas.

The thermochromic coatings, which were prepared on a polyethylene terephthalate (PET) substrate, are derived from V_0.971_F_0.029_O_2_^42^ or V_0.99_W_0.01_O_2_^43^ nanoparticles dispersed in polyurethane. These nanoparticles were produced by complex hydrothermal reactions. Here, it should be mentioned that the F-doping and W-doping of these nanoparticles resulted in the required reduction in the transition temperature, but it led also to a decrease in the modulation of the solar transmittance. The Δ*T*_sol_ value decreased from 13.1% for the coating with pure VO_2_ nanoparticles to 10.7% (see Table 2) for the coating with 2.93 at.% F-doped VO_2_ nanoparticles^42^. In case of the coating with 1 at.% W-doped VO_2_ nanoparticles^43^, the Δ*T*_sol_ value decreased to 12.7% (see Table 2) from 22.3% for the coating with pure VO_2_ nanoparticles. Note that the transition temperature *T*_tr_ = 36 °C of the high-performance thermochromic coating with the V_0.99_W_0.01_O_2_ nanoparticles (see Table 2) was determined as a mean value from the temperature of 46 °C, related to an endothermic peak, and 26 °C, related to an exothermic peak, detected using differential scanning calorimetry during the heating-up and cooling-down period, respectively.

As can be seen in Table 2, we achieved an excellent combination of the required characteristics: the lowest maximum glass temperature during the preparation of the coatings (*T*_s_)_max_ = 330 °C, an appropriate transition temperature *T*_tr_ = 20–21 °C, and *T*_lum_ up to 60% at Δ*T*_sol_ close to 6% or *T*_lum_ up to 50% at Δ*T*_sol_ above 10%. These optical properties are comparable with those achieved for the thermochromic VO_2_-based coatings which were prepared using long and too complicated chemical processes on flexible PET foils^42,43^ at a very low (*T*_s_)_max_ ≤ 100 °C, but with too high transition temperatures *T*_tr_ = 35 °C and 36 °C, respectively.

## Conclusion

High-performance thermochromic ZrO_2_/V_0.982_W_0.018_O_2_/ZrO_2_ coatings with a low transition temperature were prepared on soda-lime glass by a low-temperature scalable deposition technique which is compatible with the existing magnetron sputtering systems in glass production lines. The V_0.982_W_0.018_O_2_ layers were deposited by controlled HiPIMS of V target, combined with a simultaneous pulsed DC magnetron sputtering of W target (doping of VO_2_ by W to reduce the transition temperature to *T*_tr_ = 20–21 °C without any degradation of thermochromic properties), at a low substrate surface temperature *T*_s_ = 330 °C in an argon–oxygen gas mixture. The effective pulsed oxygen flow control of the reactive HiPIMS deposition makes it possible to utilize the enhanced energies of the ions bombarding the growing V_0.982_W_0.018_O_2_ layers for the support of the crystallization of the thermochromic VO_2_ phase in them at the low *T*_s_ = 330 °C and without any substrate bias voltage. Our design of the three-layer VO_2_-based coatings utilizes the second-order interference in two antireflection ZrO_2_ layers to increase both the luminous transmittance and the modulation of the solar transmittance. The ZrO_2_/V_0.982_W_0.018_O_2_/ZrO_2_ coatings exhibit the optical properties which are relatively close to the requirements (*T*_lum_ > 60% and Δ*T*_sol_ > 10%) for smart-window applications. For applications in large-scale systems, it is important that the presented controlled deposition of the active VO_2_-based layers can be performed also at prolonged duty cycles (up to 5%). This results in up to 5 times lower target power density in a pulse at the same deposition-averaged target power density (approximately 13 W cm^−2^ in this work). Moreover, the deposition rate of these layers is higher.

## Data Availability

All experimental deposition conditions and characterization procedures, methods and data are provided in the text. Any clarifications will be available by contacting the corresponding author.
